# Efficacy of Non-Invasive Radiofrequency-Based Diathermy in the Postoperative Phase of Knee Arthroplasty: A Double-Blind Randomized Clinical Trial

**DOI:** 10.3390/jcm10081611

**Published:** 2021-04-10

**Authors:** Manuel García-Marín, Daniel Rodríguez-Almagro, Yolanda Castellote-Caballero, Alexander Achalandabaso-Ochoa, Rafael Lomas-Vega, Alfonso Javier Ibáñez-Vera

**Affiliations:** 1 Department of Rehabilitation, University Hospital of Jaén, 23071 Jaén, Spain; mgm00049@red.ujaen.es; 2 Department of Health Sciences, University of Jaén, 23071 Jaén, Spain; dalmagro@ujaen.es (D.R.-A.); mycastel@ujaen.es (Y.C.-C.); aaochoa@ujaen.es (A.A.-O.); ajibanez@ujaen.es (A.J.I.-V.)

**Keywords:** electrophysical agents, Radiofrequency treatment, total knee replacement, total knee arthroplasty, diathermy, physical therapy modalities

## Abstract

Total knee replacement (TKR) surgery ameliorates knee function and the quality of life of patients, although 20% still experience dissatisfaction due to pain limiting their function. Radiofrequency Diathermy (MDR) has shown improvements in knee osteoarthritis and patellofemoral pain syndrome. As such, this study aims to assess the effects of MDR in the postoperative treatment of TKR patients. Forty-two participants were allocated to an experimental, placebo, or control group. For two weeks, subjects performed daily knee exercises and MDR, knee exercises and placebo MDR, or only knee exercises. Data from the Visual Analogue Scale (VAS), Timed Up-and-Go (TUG) test, Five Times Sit-to-Stand Test (FSST), Western Ontario and McMaster Universities Arthritis Index (WOMAC), physical component summary (PCS), and the mental component summary (MCS) of the SF-12 questionnaire were collected. Group-by-time interaction was significant, with favorable results in the MDR group for VAS (*p* = 0.009) and WOMAC (*p* = 0.021). No significant differences were found for TUG, FSST, PCS, or MCS (*p* > 0.05). In conclusion, the addition of MDR to therapeutic knee exercises obtained better results for knee pain than exercise alone in patients who had recently undergone TKR surgery.

## 1. Introduction

Knee osteoarthritis is an increasingly prevalent condition, with a growth of 673% expected by 2030 in the United States alone [1]. In its final stage, knee arthroplasty is usually needed to restore function and reduce pain [2]. However, over 20% of the patients who undergo this intervention report dissatisfaction with surgery results after three years [3], related to high pain scores and low functional scores [4], which usually produce kinesiophobia and avoidance behaviors [5]. Patients who undergo total knee replacement (TKR) usually report pain as the biggest barrier to recovering their usual activity [4]. Generally, drugs are used to reduce pain in these patients, particularly gabapentinoids and non-steroidal anti-inflammatory drugs [6]. However, due to addictions observed in some countries, there is special interest in reducing the prescription of medication in non-pharmacological interventions [7]. Along these lines, therapeutic exercise, electrotherapy, acupuncture, and cryotherapy have proved their effects at reducing pain and opioid consumption in TKR patients [7]. Although early physiotherapy after surgery focused on muscle strengthening and stretching is commonly recommended [8,9], evidence shows a weak relationship between dose and early response [8,10]. Based on this, clinical trials considering other rehabilitation approaches are needed [9].

Diathermy by emission of radiofrequency is a popular technique used by physiotherapists worldwide that consists of the application of a high-frequency current producing an increase in tissue temperature and metabolism [11], as well as pain reduction [12]. This treatment has shown good results in other knee conditions such as patellofemoral pain syndrome, where its addition to knee and hip strengthening home exercises resulted in better improvements than performing only the home exercises [12]. There are different types of radiofrequency diathermy based on energy transmission: capacitive-resistive [13] and capacitive-dielectric [14]. Regarding the type of application, devices can be classified as monopolar, consisting of an applicator that works as an aerial [14], and bipolar, which needs a signal transmitter and a plate to close the circuit [13]. Bipolar application is not recommended in the presence of osteosynthesis materials because metals could interfere in the electric circuit; hence, monopolar dielectric devices are considered suitable due to the lack of return element [15]. Frequencies between 400 and 900 kHz are the most used in physiotherapy practice due to their thermal effects [12,13,14,16].

Based on the positive results of monopolar radiofrequency diathermy (MDR) in patellofemoral pain syndrome and the need to improve rehabilitation results in arthroplasty patients, this study aimed to measure the efficacy of adding radiofrequency diathermy to the conventional exercise protocols for patients following TKR.

## 2. Materials and Methods

### 2.1. Study Design

A double-blind, randomized controlled trial was conducted in accordance with Good Clinical Practice and the Declaration of Helsinki. To participate in this study, all participants had to sign an informed consent form. The protocol was approved by the Ethics Investigation Committee of the Andalusian Public Health Service and registered on the Australian and New Zealand Clinical Trial Register (ANZCTR12618000258257). Data reporting complies with the Consolidated Standards of Reporting Trials (CONSORT) Statement.

### 2.2. Participants

From the 62 participants initially enrolled, 42 agreed to participate after being informed about the study and fulfilling the following inclusion criteria: (1) having undergone knee arthroplasty in the last month; (2) exhibiting pain of ≤3 points measured in a Visual Analogue Scale (VAS); (3) being between 55 and 85 years old [17]; and (4) being able to perform the measurements test. Participants that presented any of the following contraindications for treatment with MDR were excluded: tumors, use of implanted electronic devices as pacemakers, thrombophlebitis or deep venous thrombosis, and rheumatoid arthritis. Patients currently involved in a medico-legal dispute and those who have received hyaluronic acid or corticoid injections treatment were also excluded.

### 2.3. Sample Size Calculation

Calculation of the sample size was based on the data provided by Kumaran and Watson [18]. From these data, to obtain statistically significant differences among three groups with a statistical power of 80% and a confidence level of 95%, a minimum of 9 patients per group is necessary. Finally, in order to improve the statistical power, 14 patients were assigned to each group, resulting in a total of 42 subjects. This calculation was performed using the software Ene version 3.0 by the Autonomous University of Barcelona (Barcelona, Spain).

### 2.4. Randomization and Blinding

Dice rolling was used to randomize the sample and allocate participants into three groups: 1–2 for control (CG), 3–4 for experimental (EG), and 5–6 placebo (SG). One of the researchers who was not collecting data and treating the participants was in charge of delivering opaque envelopes with the group assignments. Another researcher, blinded to group designation, took the baseline and after-treatment measurements. Finally, another researcher was in charge of applying the treatment to placebo and experimental groups, using two different diathermy devices to assure blinding, as one device looked identical to the other but did not produce any signal emissions. This researcher avoided any questions from the participants about the therapy during the treatment sessions to ensure masking.

### 2.5. Measurements

All measurements were conducted by a well-trained physician with over 20 years of experience in knee management. The participants’ sociodemographic data related to age, sex, height, weight, and body mass index (BMI) were collected. The number of days since knee arthroplasty and the side of the replaced knee were also considered. Measurements were taken at baseline before the first treatment session and 30 min after the last treatment session to ensure decay of the thermal effect.

Pain was measured by VAS, which comprises a 100 mm line limited at its ends by the scores 0 (no pain) and 10 (the worst pain). This tool has shown an intraclass correlation coefficient (ICC) of 0.96 to 0.98, a standard error of measurement (SEM) of 0.03, and a minimum detectable change (MDC) of 0.08 points in knee osteoarthritis patients [19].

Several function measures were used in order to facilitate comparison with other studies: the Five Times Sit-to-Stand Test (FSST), Western Ontario and McMaster Universities Arthritis Index (WOMAC), and Timed Up-and-Go (TUG) test were used. FSST assesses participants’ mobility by timing the subjects as they stand from a sitting position five times. This test showed an inter-observer ICC of 0.995–0.999 and test–retest ICC of 0.959–0.992 (95% confidence interval) [20]. TUG evaluates mobility by having the participant get up from a chair and walk a distance of 15.2 m as fast as possible, which tests the ability to get up from a chair, walk, and maintain balance [21]. This test showed an inter-observer ICC of 0.94–0.99 and test–retest of 0.72–0.98. Moreover, a significant correlation exists between the FSST and TUG (*r* = 0.7, *p* < 0.001) [20]. Finally, the WOMAC questionnaire was used, which consists of 24 items related to knee pain, stiffness, and function. This test showed a test–retest ICC range of 0.66–0.81, and internal consistency of 0.82 for pain, 0.93 for function, and 0.81 for stiffness evaluated with Cronbach’s alpha [22].

Last, the SF-12 questionnaire was used to assess the quality of life of participants, showing an internal consistency of 0.85, measured with Cronbach’s alpha [23]. This questionnaire is the short version of the SF-36, composed of a mental component of six items (SF-12 MCS) and a physical component of another six items (SF-12 PCS).

### 2.6. Interventions

Interventions took place in the neurotraumatology facilities of Jaen Hospital. All participants followed a 30 min treatment protocol based on therapeutic knee exercises focused on strengthening and stretching the quadriceps, hamstrings, soleus, and gastrocnemius. With this aim, participants performed 10 min of active knee flexion and extension following the whole available range of motion without pain over 3 points in VAS, 10 min of walking on flat land, and 10 min of knee extension from a sitting position with a five-kilogram dumbbell placed at the subject’s foot, with one minute of rest each time participant indicated pain over 3 points in VAS. This protocol was performed for 10 therapy sessions (two weeks from Monday to Friday). In the event that severe pain appeared, the exercises were stopped until the next day.

The experimental group received ten sessions of MDR in addition to the exercises. The treatment was applied on a physiotherapy table with the participant in the supine or prone position, in a temperature-controlled room next to the rehabilitation area. An ABD Modular Device (Biotronic Advance Develops, Granada, Spain) was used to apply the MDR treatment. This therapy was applied for twelve minutes at 840 kHz modulated in 140 kHz, at 70% of pulsed emission, and at 30 V on the anterior and posterior surface of the knee. Five milliliters of almond oil were used to facilitate applicator gliding while moving over the treatment zone. Participants in the placebo group received treatment with an identical device that made the same sounds when turning on; however, the applicator did not emit any signal.

### 2.7. Statistical Analysis

Data management and statistical analysis were carried out with the Statistical Package for Social Sciences (SPSS) version 21 (SPSS Inc., Chicago, IL, USA) and with Statistical software MedCalc^®^ Statistical Software version 19.6.4 (MedCalc Software Ltd., Ostend, Belgium; https://www.medcalc.org; accessed on 8 February 2021). Data were described as mean and standard deviations for continuous variables and as frequencies and percentages for categorical variables. To verify normality and homoscedasticity, the Kolmogorov–Smirnov test and Levene’s test were used, respectively. To test between-group differences at baseline, a one-way analysis of variance and a chi-squared test were used. To test within-group differences, the Student’s *t*-test for paired samples was used. To test the effect of the therapy, a 3 × 2 mixed-model analysis of variance (ANOVA) was used to examine the effect of treatment conditions (control, placebo, or experimental) as the between-subjects variable and time (pre-treatment and post-treatment) as the within-subject variable on the dependent variables. The hypothesis of interest was the group-by-time interaction at an alpha level of 0.05. If a significant interaction was identified, pairwise Bonferroni comparisons were performed to explore between-group differences. Eta-squared and Cohen’s *d* were used to measure effect size for the group-by-time interaction and within-group differences, respectively. Eta-squared is the equivalent of the determination coefficient R^2^ for the experimental studies and could be interpreted as the proportion of the between-group differences due to the effect of the treatment. According to Cohen [24], Eta-squared can be deemed insignificant when <0.02, small if between 0.02 and 0.15, medium if between 0.15 and 0.35, and large if >0.35; and Cohen’s *d* can be interpreted as negligible (*d* < 0.2), small (*d* = 0.2 to 0.49), medium (*d* = 0.5 to 0.8), or large (*d* > 0.8).

## 3. Results

**Figure 1:**
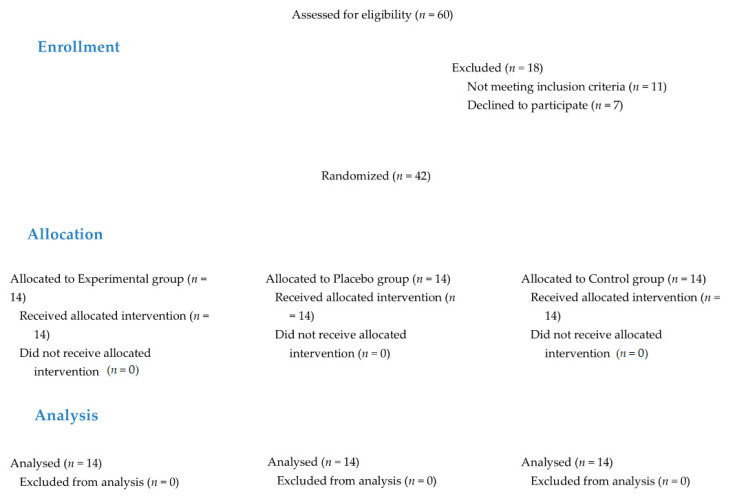
Participant flow diagram.

A final 42 patients agreed to participate in the study and complete the planned evaluations. Fourteen patients were randomized to each group (Figure 1). The characteristics and baseline measurements of each group are shown in Table 1. The groups were similar in all variables except for weight and, consequently, BMI.

In the analysis of the improvement of patients in each group, analysis of repeated measures showed that, in the control group, TUG alone showed a significant improvement with a large effect size. In the placebo group, VAS and PCS showed significant improvement with a large and medium (close to large) effect, respectively. However, in the experimental group VAS, TUG, and WOMAC showed statistically significant improvements with large effects (Table 2).

**Figure 2:**
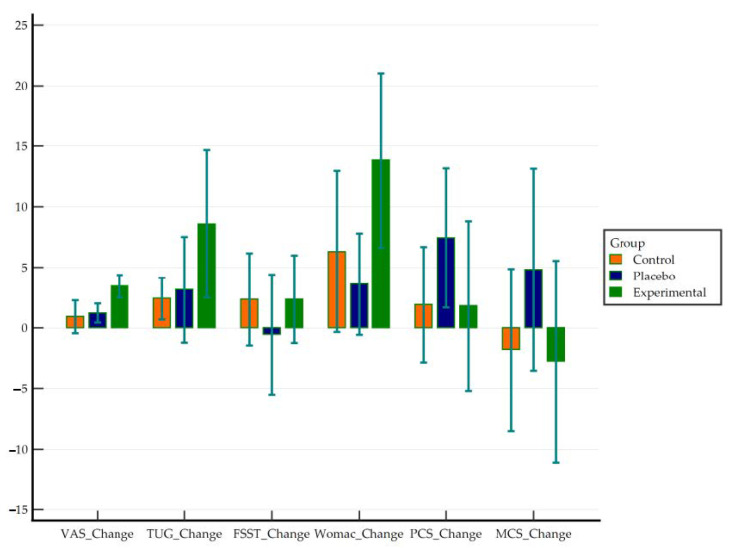
Between-group differences in change scores.

In the analysis of variance, only VAS showed a difference, favoring the experimental group vs. both placebo and control groups. WOMAC showed differences in the limits of significance, favoring the experimental vs. placebo group but not vs. control group (Figure 2). The effect, measured with Eta-squared was medium for VAS, WOMAC, and TUG (Table 3).

## 4. Discussion

The present study analyzed the effects of adding MDR treatment to the conventional therapeutic exercise program for rehabilitation following total knee replacement. The MDR therapy group showed better results regarding pain and knee function (measured with WOMAC) than both the placebo and the control groups, also showing a large effect size for both outcomes. Despite the statistically significant differences in pain indicated by the experimental group, the data should be considered with caution due to the reduced sample and the small differences with other groups. These findings are in line with those shown previously for patellofemoral pain syndrome [12] and knee osteoarthritis [18]. However, the placebo group treated with sham therapy also obtained more considerable improvements for pain than the control group, which is a recurrent finding in studies with electrotherapy devices and placebo designs [25]. More difficult to explain is the statistically significant improvement in the mental component of the SF-12 questionnaire in the placebo group, although the groups were observed to overlap in the plots. Concerning knee function, only the MDR group achieved statistically significant improvements in WOMAC (*p* = 0.021) in the between-group comparison, but not in the FSST or TUG. This could be explained by the higher complexity of these functional tests, which include not only the knee but also the hip, ankle, and balance. Nonetheless, data shows that the improvements in knee function of TKR patients could be more a consequence of therapeutic exercise, although, based on the WOMAC results, MDR therapy could also play a significant role.

Following TKR, patients usually report pain as being the highest inconvenience when recovering their usual activity. According to this study, MDR treatment results in similar pain reduction (34 mm in VAS) to gabapentinoids (from 19 to 4 mm in VAS) and non-steroidal anti-inflammatory drugs/COX-2 inhibitors (around 30 mm in VAS) [6]. This is very relevant as it could be considered an alternative approach to prevent opioid addiction and be used for patients with hypertension [7]. When compared with other alternatives to drug treatment, MDR seems to obtain larger improvements than transcutaneous electrical nerve stimulation (21 mm in VAS), acupuncture (16 mm in VAS reduction), and cryotherapy (from 5 to 10 mm in VAS) [7]. However, all these comparisons are unreliable, as each treatment’s results were measured according to different timelines (MDR results were measured after two weeks of treatment, whereas acupuncture and cryotherapy results were measured 48 h after intervention).

To our knowledge, this is the first study in which patients are treated with MDR following total knee. Other studies could be considered similar, such as the one by Kumaran and Watson (2019), who treated knee osteoarthritis patients, observing almost 10 mm of improvement in VAS for the experimental group when compared to control and placebo [18]. Another study analyzing the effects of MDR on knee pain is by Albornoz-Cabello et al. (2020), who obtained 49 mm of improvement in VAS for patients with patellofemoral pain syndrome [12]. These results cannot be compared with the ones obtained in the present study due to the different pathologies; however, it can be interpreted as a common point of pain relief, which inversely correlates with the degeneration level of the knee.

Concerning the clinical importance of our results, the minimal clinically important difference (MCID) for VAS in studies involving knee arthroplasty has been calculated at 15 mm [26]. In our study, both the control group (9 mm) and the placebo group (12 mm) obtained improvements below the MCID, while the experimental group did obtain a higher improvement (34 mm). However, the difference between the active therapy group compared to the placebo and control groups was 11 and 12 mm respectively, which is below the MCID for this condition and thus the findings must be considered with caution.

Regarding knee function, the benefits of therapeutic exercise are clear and are the reason it is always included as a recommendation in clinical guidelines [8,9]. In fact, progressive strengthening of knee muscles shows greater effects in function than conventional care [27] and reduces the number of outcomes in TKR patients [28]. However, the lack of a strong consensus regarding the type of exercises, the load, and the number of repetitions increases the heterogeneity of studies and the difficulty to establish comparisons [10]. According to the present study’s data, the addition of MDR to therapeutic exercise obtained more considerable improvements in knee function than therapeutic exercise treatment alone. This may be explained by the pain reduction that MDR produces in TKR patients or the blood flow increment produced by the deep heating of the tissue [11], which enhances the oxygen availability in the local tissues.

For any chronic condition, the focus is always on the patient’s quality of life and, accordingly, some authors also point to the importance of quality of life in TKR patients [2]. The present study found no important short-term differences in quality of life measured by the physical function of the SF-12 after TKR surgery. However, statistically significant differences related to the knees’ physical daily function were obtained in the MDR group. This disagreement could support the hypothesis that patients need more time to become aware of their improvements, as measurements were performed only a few weeks after surgery.

This study has several limitations. Firstly, follow-up with participants was planned but not carried out due to the pandemic situation. Secondly, despite the sample size calculation, we believe that a larger sample size could better clarify some unexpected phenomena such as the improvements in the mental component of SF-12 of the placebo group. Finally, the masking of the researcher applying the treatments would have improved the quality of this trial. Further studies are needed to assess the role of MDR in functional knee improvements.

## 5. Conclusions

The addition of non-invasive radiofrequency-based diathermy (MDR) to therapeutic exercise appears to reduce pain in patients who have recently undergone total knee replacement surgery. However, the role of MDR in knee function is not clear, and further studies are required.

## Figures and Tables

**Table 1:** Sociodemographic characteristics and baseline differences.

		All Participants		Control		Placebo		Experimental		*p*-Value **
Gender	Male	15	35.7%	5	35.7%	4	28.6%	6	42.9%	0.733
	Female	27	64.3%	9	64.3%	10	71.4%	8	57.1%	
TKR Side	Left	20	47.6%	3	21.4%	8	57.1%	9	64.3%	0.052
	Right	22	52.4%	11	78.6%	6	42.9%	5	35.7%	
		**M**	**SD**	**M**	**SD**	**M**	**SD**	**M**	**SD**	
Age	(years)	69.36	7.16	69.71	6.89	69.29	8.33	69.07	6.68	0.973
Weight	(kg)	82.68	15.79	90.18	15.13	83.21	18.87	74.64	8.40	0.029 *
Height	(m)	1.58	0.09	1.60	0.07	1.57	0.10	1.57	0.10	0.611
BMI		33.07	4.76	35.17	4.40	33.46	5.09	30.58	3.84	0.032 *
Days since surgery		20.24	13.86	20.50	14.55	23.50	16.92	16.71	9.11	0.441
VAS		5.67	2.04	5.18	2.04	5.29	1.77	6.54	2.15	0.149
TUG		16.98	10.39	13.89	3.73	15.90	11.02	21.17	13.21	0.161
FSST		16.35	5.84	17.54	5.17	14.67	5.44	16.83	6.82	0.411
WOMAC		27.73	16.73	26.29	9.90	26.15	14.49	30.85	24.04	0.726
PCS		42.50	4.90	41.95	4.57	44.72	4.28	40.15	5.37	0.082
MCS		37.21	8.52	39.08	8.32	37.39	9.87	34.05	6.47	0.396

BMI: body mass index; VAS: Visual Analogue Scale; TUG: Timed Up-and-Go test; FSST: Five Times Sit-to-Stand Test; WOMAC: Western Ontario and McMaster Universities Arthritis Index; PCS: physical component of SF-12 questionnaire; MCS: mental component of SF-12 questionnaire; M: mean; SD: standard deviation; TKR: total knee replacement. * *p* < 0.05; ** *p*-value obtained by chi-squared test for categorical variables and by one-way analysis of variance for continuous variables.

**Table 2:** Descriptive data of change scores. Statistical significance and effect size of the within-group differences.

CONTROL	Mean	SD	SE	Lower	Upper	*p*-Value	Cohen’s *d*	Effect
VAS	0.92	2.35	0.63	−0.43	2.28	0.166	0.39	Small
TUG	2.42	2.87	0.79	0.69	4.15	0.01 *	0.84	Large
FSST	2.36	6.61	1.77	−1.46	6.17	0.205	0.36	Small
WOMAC	6.31	11	3.05	−0.34	12.95	0.061	0.57	Medium
PCS	1.91	7.91	2.19	−2.87	6.69	0.401	0.24	Small
MCS	−1.8	11.07	3.07	−8.49	4.88	0.567	−0.16	Negligible
**PLACEBO**	**Mean**	**SD**	**SE**	**Lower**	**Upper**	***p*-Value**	**Cohen’s *d***	**Effect**
VAS	1.21	1.37	0.37	0.42	2	0.006 **	0.89	Large
TUG	3.15	7.58	2.03	−1.23	7.53	0.144	0.42	Small
FSST	−0.54	8.58	2.29	−5.5	4.41	0.816	−0.06	Negligible
WOMAC	3.62	6.93	1.92	−0.58	7.81	0.085	0.52	Medium
PCS	7.43	9.52	2.64	1.68	13.18	0.016 *	0.78	Medium
MCS	4.81	13.8	3.83	−3.52	13.15	0.232	0.35	Small
**EXPERIMENTAL**	**Mean**	**SD**	**SE**	**Lower**	**Upper**	***p*-Value**	**Cohen’s *d***	**Effect**
VAS	3.44	1.6	0.43	2.52	4.37	0.000 ***	2.15	Large
TUG	8.59	10.54	2.82	2.5	14.67	0.009 **	0.81	Large
FSST	2.36	6.27	1.67	−1.26	5.98	0.183	0.38	Small
WOMAC	13.83	11.34	3.27	6.63	21.04	0.001 **	1.22	Large
PCS	1.81	8.38	2.96	−5.2	8.82	0.561	0.22	Small
MCS	−2.77	9.93	3.51	−11.07	5.54	0.456	−0.28	Small

VAS: Visual Analogue Scale; TUG: Timed Up-and-Go test; FSST: Five Times Sit-to-Stand Test; WOMAC: Western Ontario and McMaster Universities Arthritis Index; PCS: physical component of SF-12 questionnaire; MCS: mental component of SF-12 questionnaire; M: mean; SD: standard deviation; SE: standard error; Cohen’s *d*: effect size calculated with Cohen’s *d*. * *p* < 0.05; ** *p* < 0.01; *** *p* < 0.001.

**Table 3:** Post-treatment scores, significance, and effect size of the group-by-time interaction.

Variable	Control		Placebo		Experimental		*p*-Value	ETA^2^
	M	SD	M	SD	M	SD		
VAS	4.25	2.64	4.15	1.68	3.04	1.95	0.009 **	0.277
TUG	11.88	2.19	13.09	5.34	13.50	6.20	0.092	0.152
FSST	14.56	1.97	15.59	4.82	14.89	5.24	0.456	0.053
WOMAC	21.42	9.31	22.54	13.30	21.38	20.74	0.021 *	0.235
PCS	39.18	4.38	37.29	10.88	37.56	5.97	0.276	0.085
MCS	41.81	7.55	32.57	9.52	37.49	7.46	0.219	0.099

VAS: Visual Analogue Scale; TUG: Timed Up-and-Go test; FSST: Five Times Sit-to-Stand Test; WOMAC: Western Ontario and McMaster Universities Arthritis Index; PCS: physical component of SF-12 questionnaire; MCS: mental component of SF-12 questionnaire; M: mean; SD: standard deviation; ETA^2^: effect size. * *p* < 0.05; ** *p* < 0.01.

## Data Availability

The data of this study is available under request to the corresponding author.
